# Supplementary material for: Oxidized nucleotide insertion by pol β confounds ligation during base excision repair
Source: Nat Commun. 2017 Jan 9;8:14045. doi: 10.1038/ncomms14045 (PMC5228075; doi:10.1038/ncomms14045)
Supplement: Supplementary Information — Supplementary Figures and Supplementary Tables [file ncomms14045-s1.pdf]

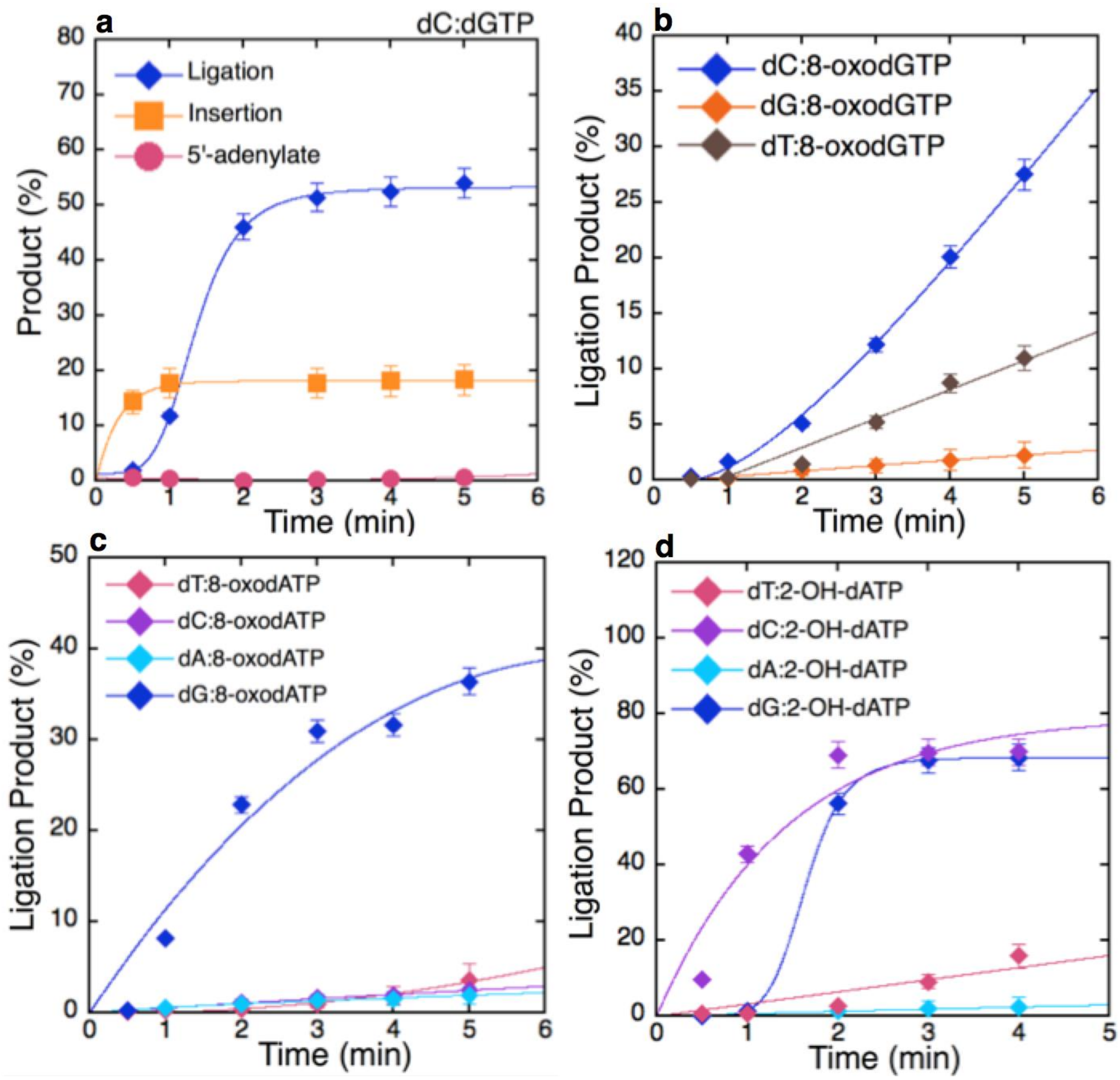

Supplementary Fig. 1: Ligation comparison. Time-dependent changes in the products of ligation and insertion for dC:dGMP (**a**) Plots show template base-dependent changes in ligation products for the substrates C^{gap}, G^{gap}, and T^{gap} (**b**), oxidized nucleotide and template base-dependent changes of 8-oxodATP (**c**) and 2-OH-dATP (**d**) for the substrates A^{gap}, C^{gap}, G^{gap}, and T^{gap}. The data represent mean values with the standard deviation from three independent experiments.

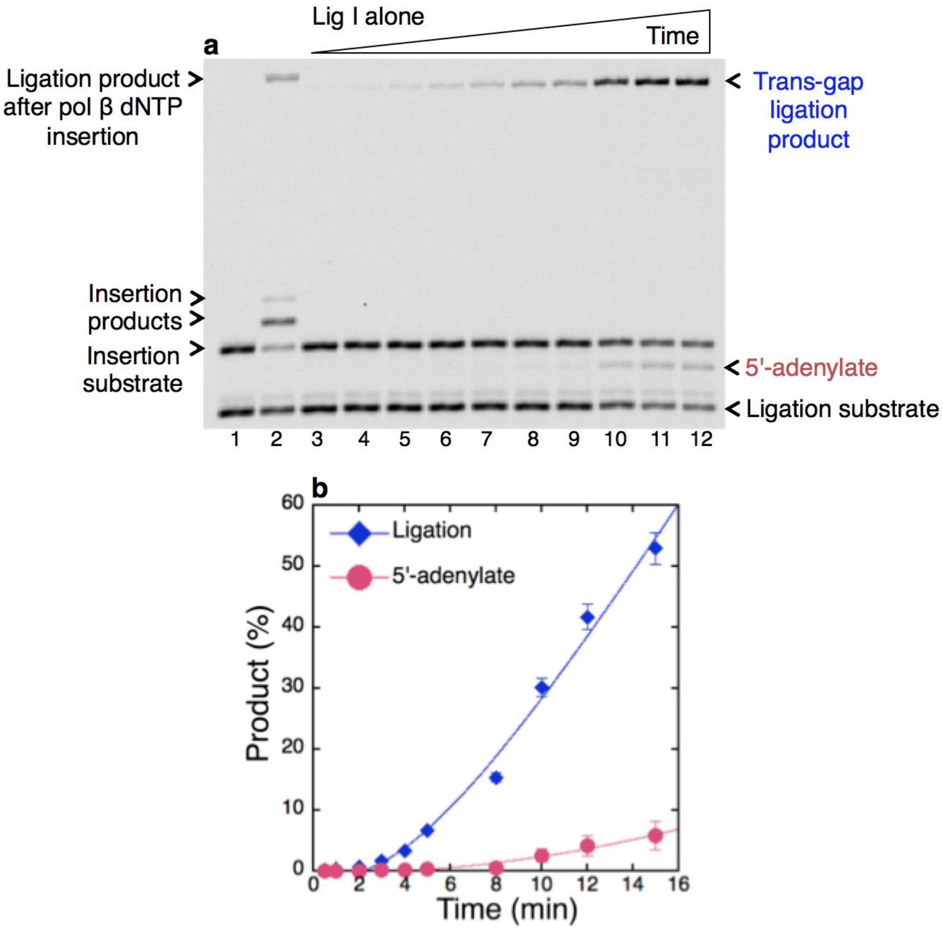

Supplementary Fig. 2: Ligation failure in a control reaction with ligase alone. (**a**) Lane 1 is the minus enzyme control, and lane 2 is the product of coupled BER reaction including Lig I, pol β, and dGTP used as a marker. Lanes 3-12 are the products of ligation reaction including Lig I alone and correspond to time points of 0.5, 1, 2, 3, 4, 5, 8, 10, 12, and 15 min. (**b**) Quantification of the data presented in panel a. The data represent mean values with the standard deviation from three independent experiments.

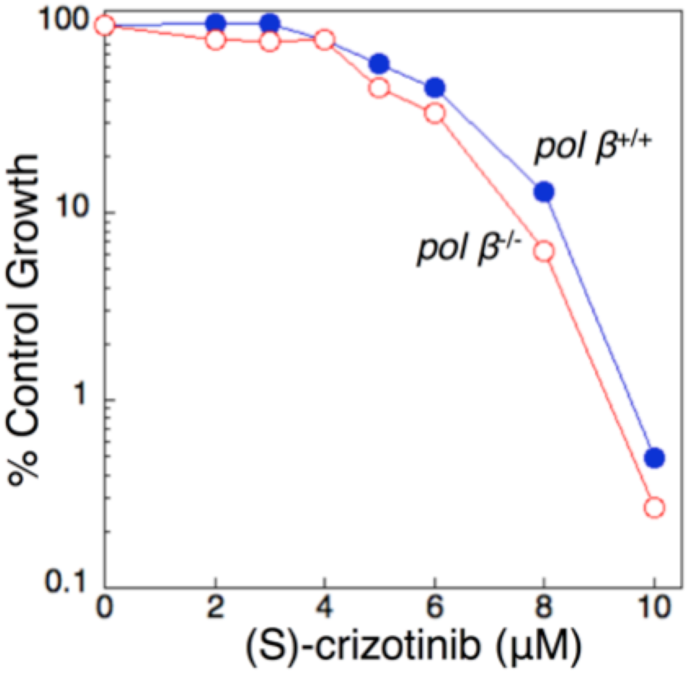

Supplementary Fig. 3: Cytotoxicity of continuous (S)-crizotinib alone (0-10 µM) in *pol β*$^{+/+}$ and *pol β*$^{-/-}$ cells.

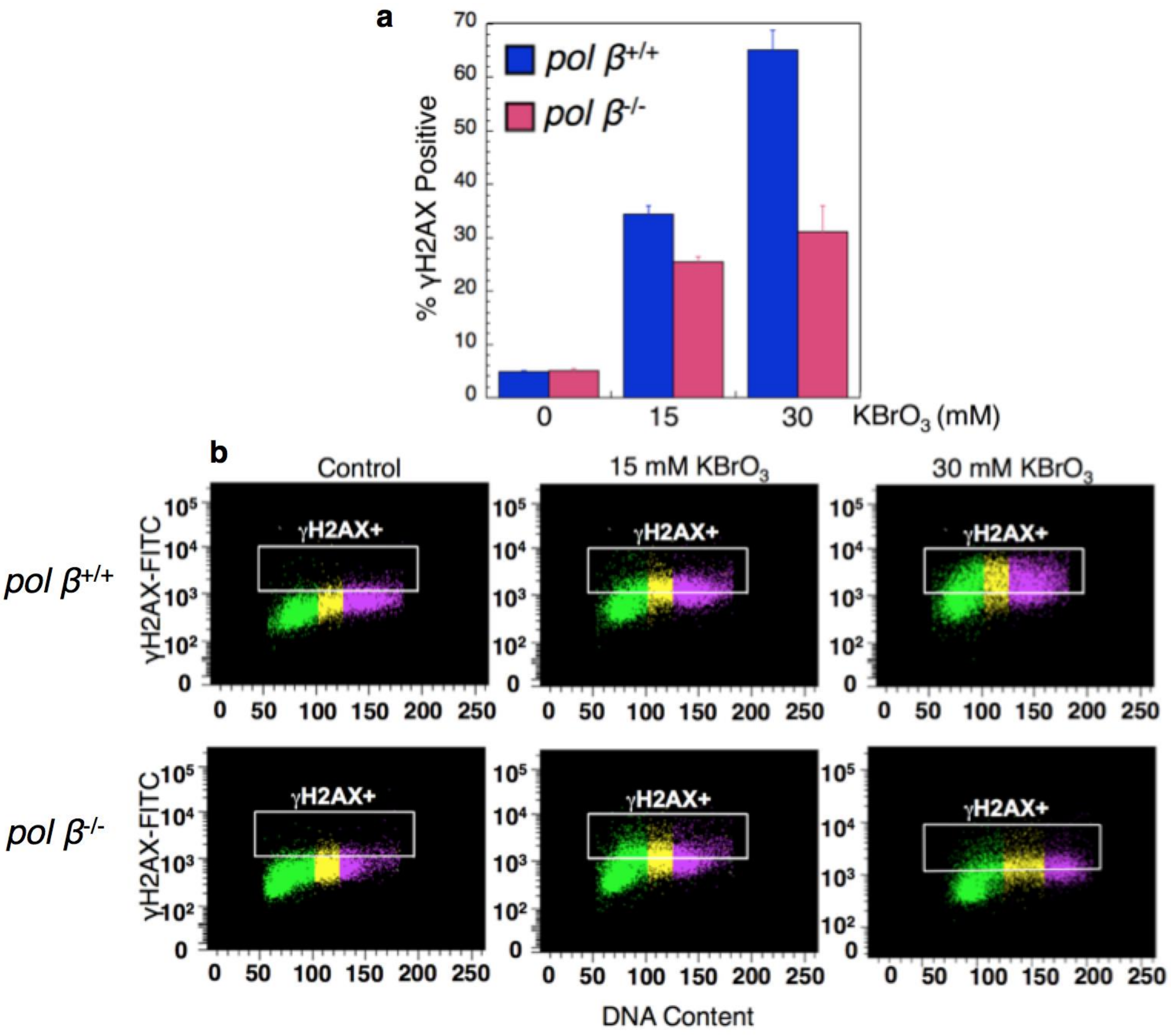

Supplementary Fig. 4: Flow cytometric quantification of γH2AX staining and cell cycle of mouse embryonic fibroblasts following oxidative stress. (**a**) % γH2AX staining of *pol* $\beta^{+/+}$ and *pol* $\beta^{-/-}$ cells corresponding to control and 4 h after treatment with $KBrO_3$ (15 and 30 mM). (**b**) The phases of the cell cycle are colored as G1 (green), S (yellow), and G2/M (pink). The boxed populations represent γH2AX positive cells. The proportions of cells in each phase of the cell cycle are presented in Supplementary Table 3. The data represent mean values from three independent experiments.

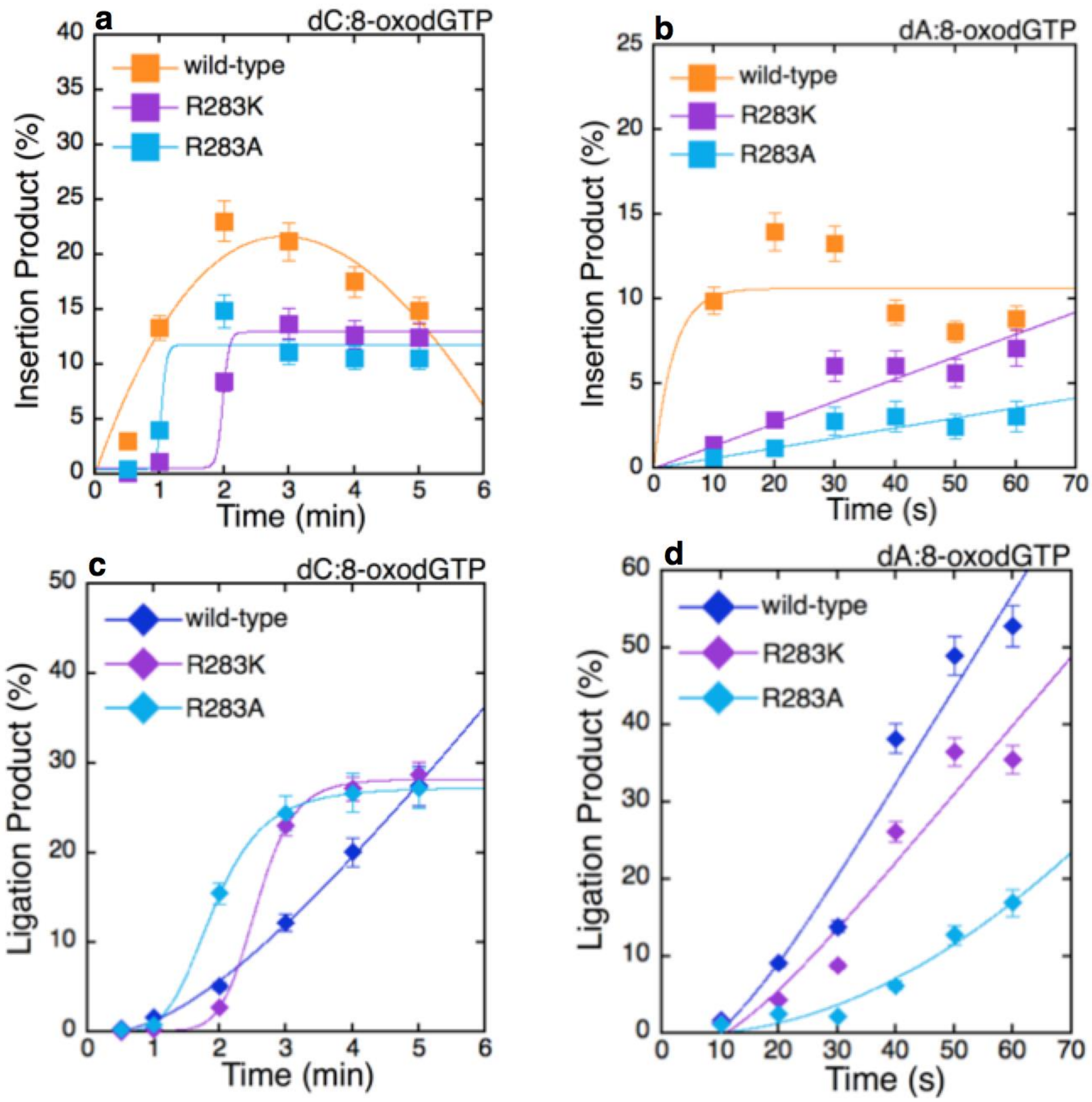

Supplementary Fig. 5: Comparison of insertion and ligation for pol β active site mutants R283A and R283K. Plots show time-dependent changes in the products of insertion for the substrate C^{gap} (**a**), A^{gap} (**b**) and ligation for the substrate C^{gap} (**c**) and A^{gap} (**d**). The data represent mean values with the standard deviation from three independent experiments.

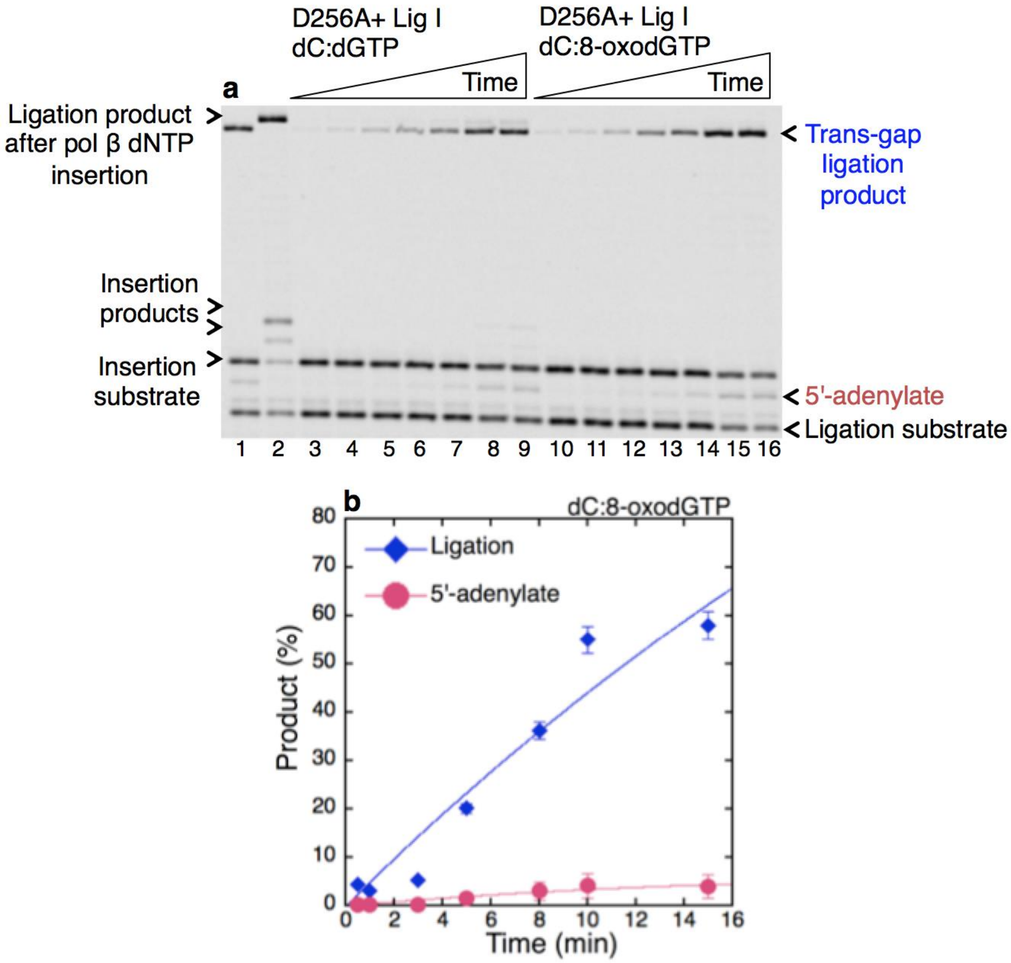

Supplementary Fig. 6: Ligation failure in a control reaction with pol β D256A mutant. (**a**) Lane 1 is the reaction including Lig I alone, and lane 2 is the product of coupled BER reaction including Lig I, pol β, and dGTP used as a marker. Lanes 3-9 and 10-16 are the reaction products of coupled BER reaction including pol β D256A mutant and Lig I in the presence of dGTP and 8-oxodGTP, respectively, and the reactions correspond to time points of 0.5, 1, 3, 5, 8, 10 and 15 min. (**b**) Quantification of the data for dC:8-oxodGTP presented in panel a. The data represent mean values with the standard deviation from three independent experiments.

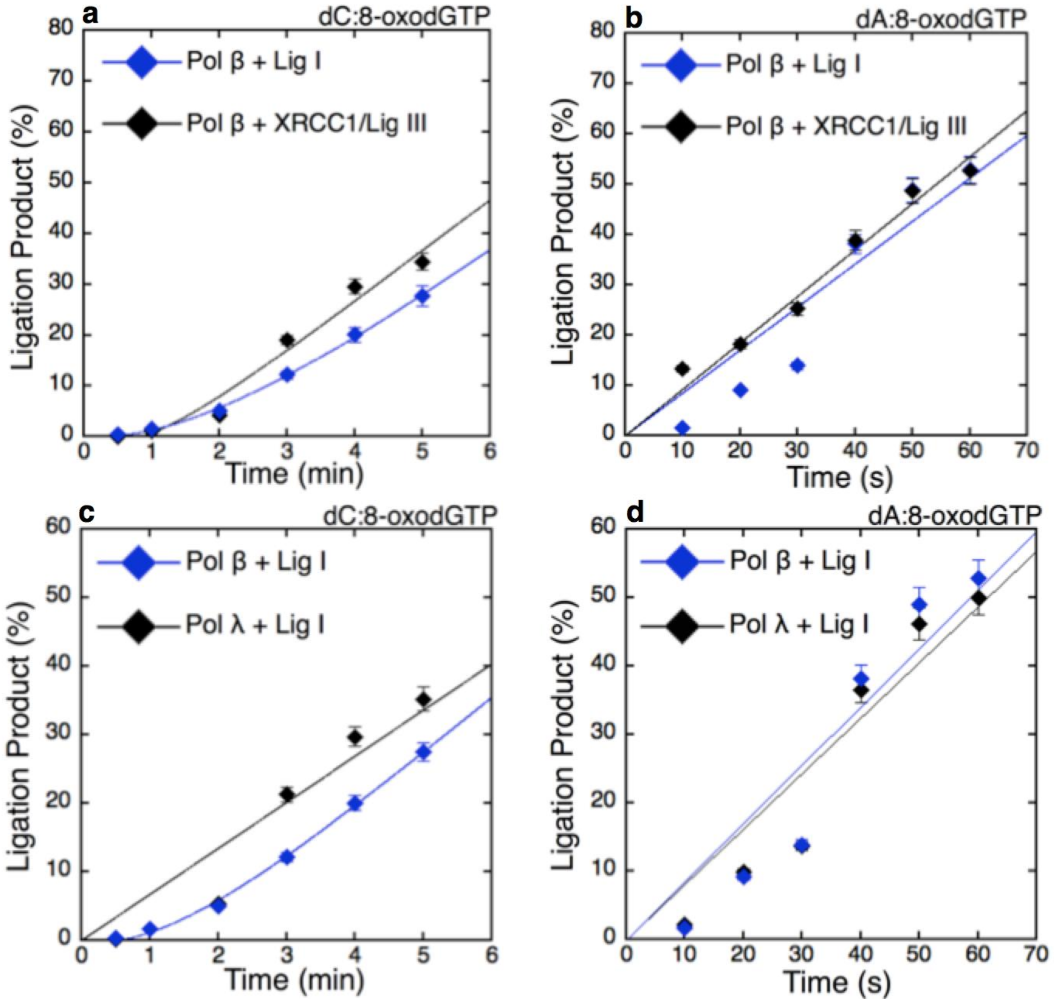

Supplementary Fig. 7: Comparison of ligation for XRCC1/Lig III and pol λ. Plots show time-dependent changes in the products of ligation for Lig I versus XRCC1/Lig III complex for the substrates C^{gap} (**a**) and A^{gap} (**b**) and for pol β versus pol λ for the substrates C^{gap} (**c**) and A^{gap} (**d**). The data represent mean values with the standard deviation from three independent experiments.

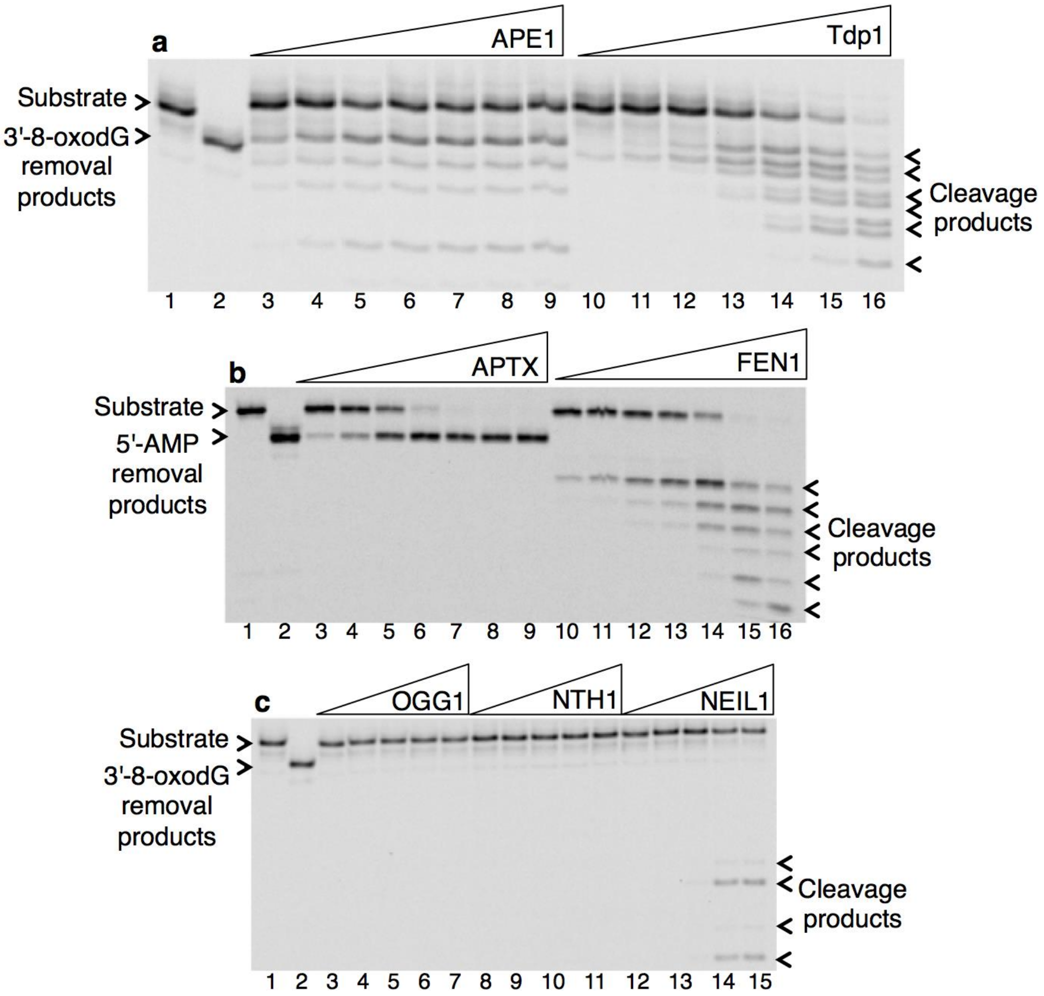

Supplementary Fig. 8: Role of 5'- and 3'-end processing enzymes in repair of blocked BER intermediate. (**a**) Lane 1 is the minus enzyme control, and lane 2 is the oligonucleotide without 3'-8-oxoG used as a marker. Lanes 3-9 and 10-16 are the reaction products observed in the concentration range (10-500 nM) of APE1 and Tdp1, respectively. (**b**) Lane 1 is the minus enzyme control, and lane 2 is the oligonucleotide without 5'-AMP used as a marker. Lanes 3-9 and 10-16 are the reaction products observed in the concentration range (10-500 nM) of APTX and FEN1, respectively. (**c**) Lane 1 is the minus enzyme control, and lane 2 is the oligonucleotide without 3'-8-oxoG used as a marker. Lanes 1-5, 6-10, and 11-15 are the reaction products observed in the concentration range (10-500 nM) of OGG1, NTH, and NEIL1, respectively.

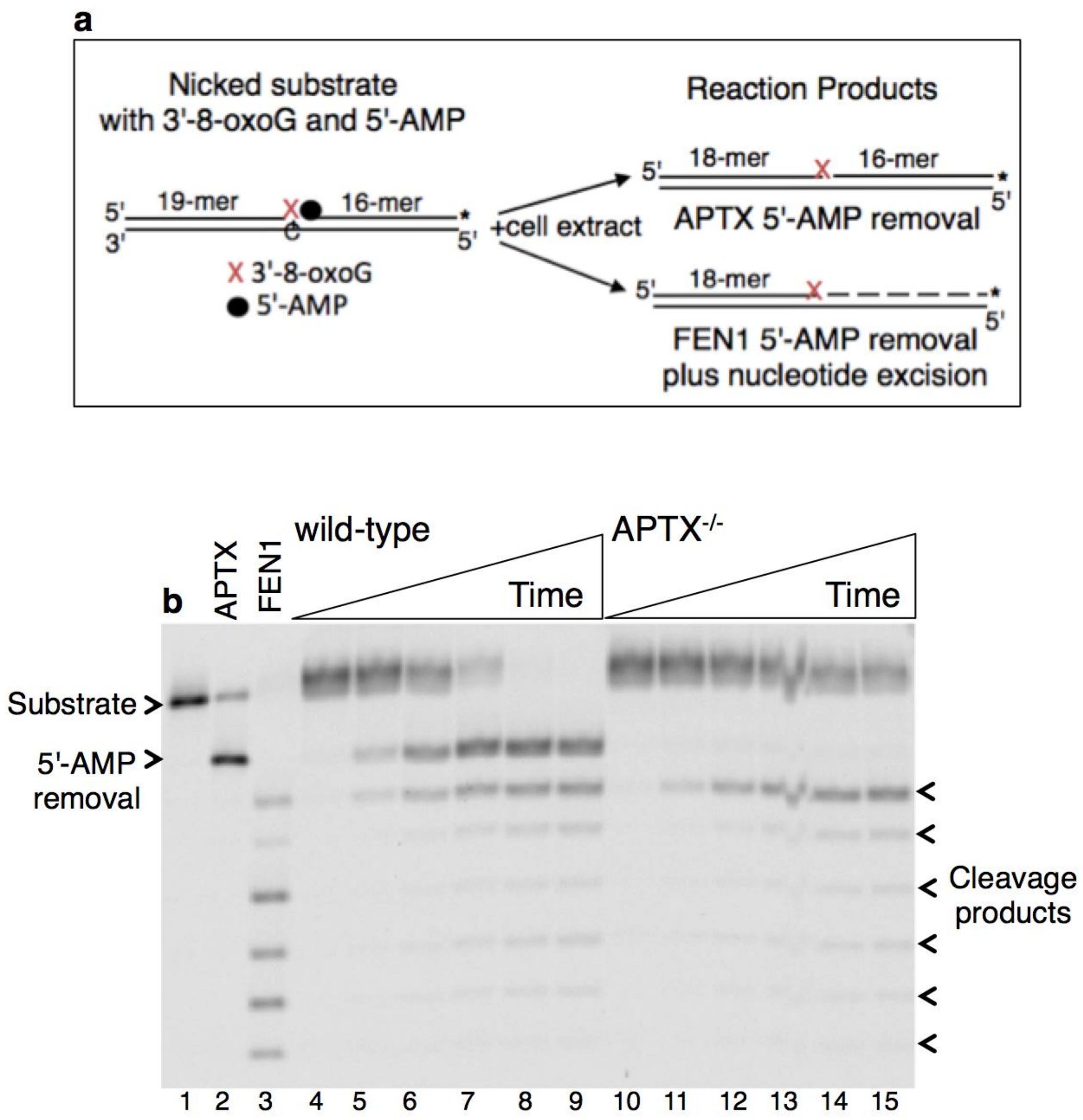

Supplementary Fig. 9: Processing of the 3'-8-oxoG and 5'-AMP-including BER intermediate by cell extracts from DT40 cells. (**a**) Illustrations of nicked DNA substrate (Substrate 2, Supplementary Table 1) and reaction products observed. (**b**) Lane 1 is the minus enzyme control, and lanes 2 and 3 are reference reaction products of purified enzymes APTX and FEN1, respectively. Lanes 4-9 and 10-15 are the reaction products observed in cell extracts from wild-type and APTX-deficient cells, and correspond to time points 5, 10, 15, 20, 25, 30 min, respectively.

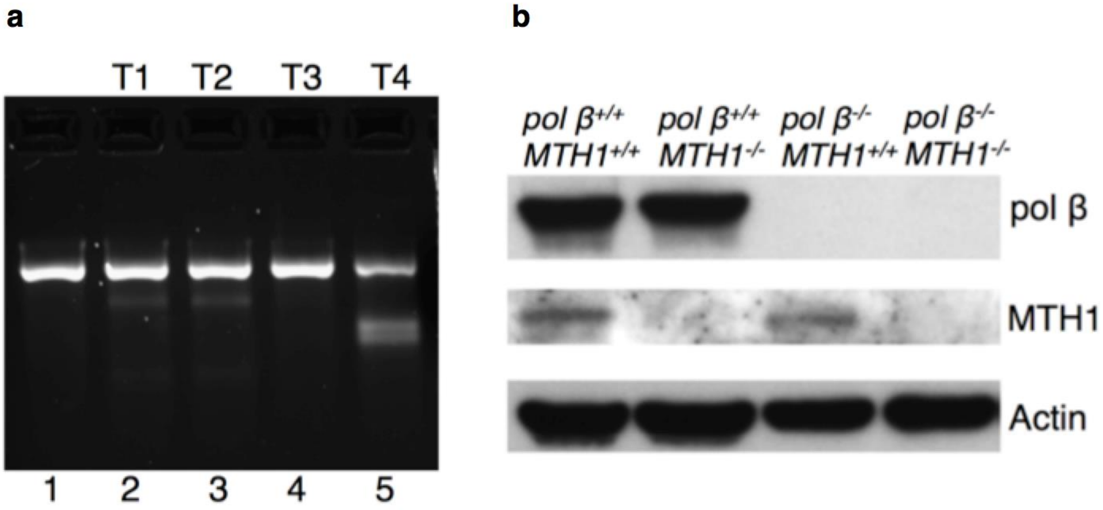

Supplementary Fig. 10: Construction of MTH1 knockout cell lines using CRISPR-eCas9 system. (**a**) Lane 1 represents cells infected with empty vector Lenti-eSpCas9 (control), and lanes 2-5 (T1-T4; Targets 1-4) represent cells infected with lentivirus containing RNA-guided eSpCas9 nuclease and different sgRNAs targeting different regions in the mouse *MTH1* gene. T4 shows higher genome editing capacity compared to the other three guide sequences and is used for further stable cell line selection. (**b**) Western blot verification of stable cell lines; *MTH1*$^{-/-}$ was isolated using Target 4.

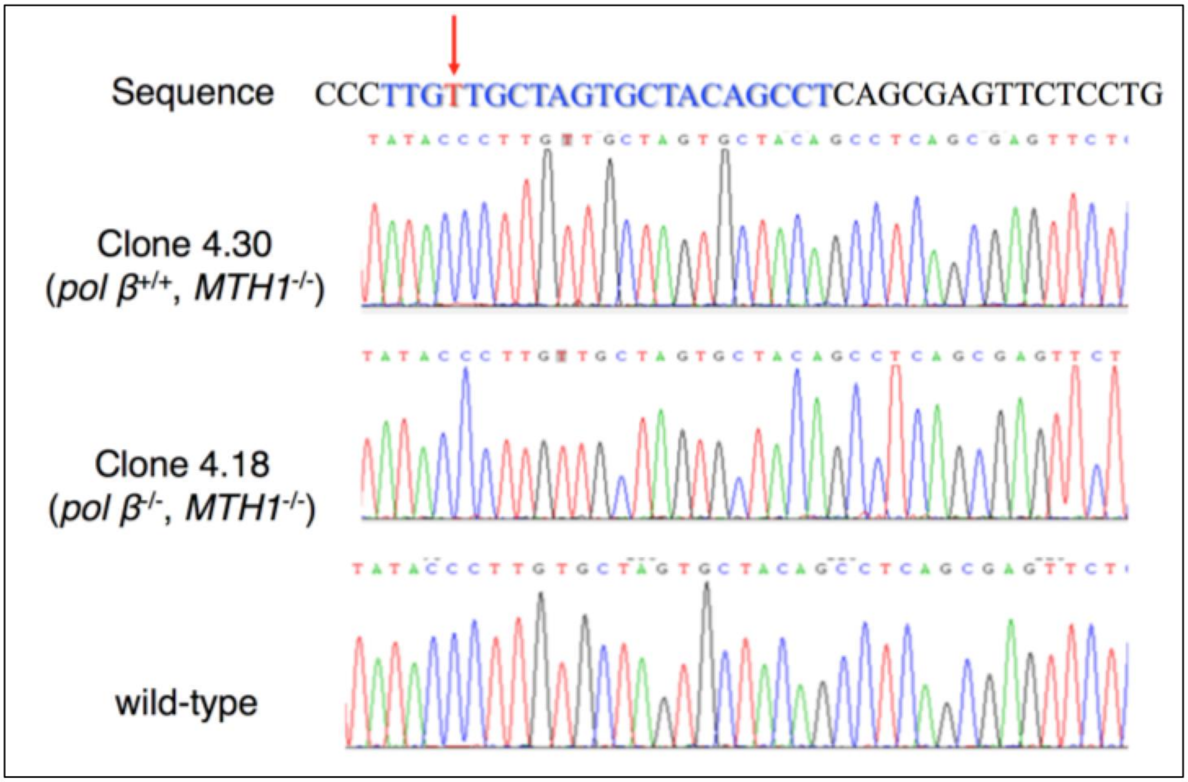

Supplementary Fig. 11: Sequencing results of MTH1 knockout cell lines. Two *MTH1*$^{-/-}$ stable cell lines were sequenced around the designed targeting region of the *MTH1* gene. Both clones showed the same mutation type in which one nucleotide T was inserted between the position 28 and 29 of *MTH1* cDNA. The designed target region in *MTH1* gene is illustrated as blue letters and the inserted nucleotide is shown in red resulting in a frameshift and the creation of a premature stop codon.

Substrate	Sequence
A^{gap}	FAM-5’-AACATGGGCGGCATGAAAT AATGCCCATCCTCACCA-3’-FAM<br>3’-TTGTACCCGCCGTACTTTAATTACGGGTAGGAGTGGT-5’
T^{gap}	FAM-5’-AACATGGGCGGCATGAAAT AATGCCCATCCTCACCA-3’-FAM<br>3’-TTGTACCCGCCGTACTTTATTTACGGGTAGGAGTGGT-5’
C^{gap}	FAM-5’-AACATGGGCGGCATGAAAT AATGCCCATCCTCACCA-3’-FAM<br>3’-TTGTACCCGCCGTACTTTACTTACGGGTAGGAGTGGT-5’
G^{gap}	FAM-5’-AACATGGGCGGCATGAAAT AATGCCCATCCTCACCA-3’-FAM<br>3’-TTGTACCCGCCGTACTTTAGTTACGGGTAGGAGTGGT-5’
A^{nick}	5’-CATGGGCGGCATGAACCXGAGGCCCATCCTCACC-3’-FAM<br>3’-GTACCCGCCGTACTTGGACTCCGGGTAGGAGTGG-5’
T^{nick}	5’-CATGGGCGGCATGAACCXGAGGCCCATCCTCACC-3’-FAM<br>3’-GTACCCGCCGTACTTGGTCTCCGGGTAGGAGTGG-5’
C^{nick}	5’-CATGGGCGGCATGAACCXGAGGCCCATCCTCACC-3’-FAM<br>3’-GTACCCGCCGTACTTGGCCTCCGGGTAGGAGTGG-5’
G^{nick}	5’-CATGGGCGGCATGAACCXGAGGCCCATCCTCACC-3’-FAM<br>3’-GTACCCGCCGTACTTGGGCTCCGGGTAGGAGTGG-5’
1	AMP<br>FAM-5’-ACATGGGCGGCATGAACCXGAGGCCCATCCTCACC-3’<br>3’-TGTACCCGCCGTACTTGGCCTCCGGGTAGGAGTGG-5’
2	AMP<br>5’-ACATGGGCGGCATGAACCXGAGGCCCATCCTCACC-3’-FAM<br>3’-TGTACCCGCCGTACTTGGCCTCCGGGTAGGAGTGG-5’

Supplementary Table 1: The gapped and nicked DNA substrates used in this study. FAM indicates presence of a fluorescent tag, and X represents 8-oxoG base. The base underlined in sequence indicates template base A, T, C or G.

Primer	Sequence	Purpose
eSpCas9-F	CCGGTtctagaGCGCTGCCACCATGGACTATAAGGACCA	amplify the expression cassette of eSpCas9 (introduced XbaI and BamHI sites were shown in lowcase)
eSpCas9-R	GGGGTggatccCTTTTTCTTTTTTGCCTGGC	
mMTH1-T1F	CACCGGGCCGCTGGAATGGCTTCGG	clone sgRNA of target 1 into lenti-eSpCas9 vector
mMTH1-T1R	AAACCCGAAGCCATTCCAGCGGCCC	
mMTH1-T2F	CACCGTGGCCGCTGGAATGGCTTCG	clone sgRNA of target 2 into lenti-eSpCas9 vector
mMTH1-T2R	AAACCGAAGCCATTCCAGCGGCCAC	
mMTH1-T3F	CACCGTTTGGTGCTGGCCGCTGGAA	clone sgRNA of target 3 into lenti-eSpCas9 vector
mMTH1-T3R	AAACTTCCAGCGGCCAGCACCAAAC	
mMTH1-T4F	CACCGAGGCTGTAGCACTAGCACAA	clone sgRNA of target 4 into lenti-eSpCas9 vector
mMTH1-T4R	AAACTTGTGCTAGTGCTACAGCCTC	
mMTH1-1F1	ACCATCCACCTCTCCCAGAA	amplify part of the exon 1 of *MTH1* gene for SURVEYOR assay
mMTH1-1R1	CTCTCTTGGGCTTCATCCCC	
hU6-F	GAGGGCCTATTTCCCATGATT	Sanger sequencing of lenti-eSpCas9 about the sgRNA guide sequences
mMTH1-seq	CCTGGCCACAACAGAACTCA	Sanger sequencing of exon1 of *MTH1* gene

Supplementary Table 2: Oligonucleotides and primers used in this study for *MTH1* gene deletion.

pol β$^{+/+}$

	Control	15 mM $KBrO_3$	30 mM $KBrO_3$
γH2AX+	4.85	34.5	65.2
G1	47.7	45.1	47.4
S	17.1	18.8	18.7
G2/M	36.3	37.7	35.1

pol β$^{-/-}$

	Control	15 mM $KBrO_3$	30 mM $KBrO_3$
γH2AX+	5.1	25.4	31.1
G1	61.1	54.0	60.6
S	24.6	22.4	19.8
G2/M	16.1	25.4	20.1

Supplementary Table 3: The percentage of cells in each phase of the cell cycle and γH2AX+ cells in control and $KBrO_3$-treated cells. Flow cytometric images and quantification of γH2AX staining of mouse embryonic fibroblasts after oxidative stress are presented in Supplementary Fig. 4. Gates were set using contour plots and determination of center points of G1 and G2/M for reference with the population in the middle determined as S phase.
